# Preparing for life beyond sport: dual career awareness, developmental environment, and future orientation in adolescent handball

**DOI:** 10.3389/fpsyg.2026.1829338

**Published:** 2026-05-25

**Authors:** József Bognár, Balázs Fügedi, Barbara Bácskay-Abonyi, Zsolt Szakály, István Juhász, István Simon

**Affiliations:** 1 Institute of Sport Science, Eszterházy Károly Catholic University, Eger, Hungary; 2 Sport and Health Science Research Group, Eszterházy Károly Catholic University, Eger, Hungary; 3 Apáczai Csere János Faculty of Humanities Education and Social Sciences, Széchenyi István University, Györ, Hungary; 4 Benedek Elek Faculty of Pedagogy, University of Sopron, Sopron, Hungary

**Keywords:** career adaptability, holistic athlete development, psychosocial competencies, student-athletes, talent development environment, transition out of sport

## Abstract

Adolescence represents a crucial developmental stage for career construction and future orientation, particularly among young athletes who demonstrate a high level of commitment to elite sport. However, empirical evidence regarding the preparatory phase of dual career during this stage remains limited. The aim of the present study was to explore how the personality traits of secondary school handball players, their sport career aspirations, their parallel educational ambitions, and their perceived developmental environment contribute to their career construction, and how these factors are reflected in the coaches' perceptions. The sample consisted of 507 registered secondary school handball players (*M* = 16.36 ± 2.19 years) and 279 coaches (*M* = 44.60 ± 12.74 years). Data were collected using the Erasmus+ DC4AC questionnaire. Descriptive statistical indicators and independent two sample *t*-tests were applied during the analysis. The results indicate that athletes perceive themselves as curious, confident, and relatively prepared for life after sport, and at the same time reporting lower levels of concern regarding their future and dual career issues. Respondents perceive developmental practices primarily as oriented toward physical and sport-specific development, while psychological, academic, and social dimensions receive less emphasis. Significant perceptual differences were identified between athletes and coaches, particularly regarding preparedness and holistic development and gender differences revealed additional nuances in future orientation and developmental priorities (*p* < 0.05). The findings highlight the importance of a holistic and institutionally supported dual career approach in adolescent talent development sport, which may strengthen career adaptability and reduce risks associated with a one-sided athletic identity.

## Introduction

Adolescence represents a particularly important developmental stage for exploring career opportunities and establishing the foundations of an individual life trajectory. During this period, young people select activities and environments in which their future career aspirations appear attainable, based on their interests, perceived competencies, personal values, and developmental needs (Savickas, 2013). Consequently, the preparation for career-related decisions and the development of long-term commitment are considered central developmental tasks for adolescent athletes (Staff et al., 2009; Super et al., 1996).

Within this developmental context, many successful adolescent athletes aspire to pursue a career in elite sport. However, such a pathway requires substantial temporal, physical, and psychological investment from an early age. Accordingly, the secondary school years are often conceptualized as the investment stage of the athletic career (Stambulova et al., 2015), during which athletes increasingly dedicate themselves to high-level training and competition.

At the same time, this intensified commitment to sport carries potential risks. A lack of sustained commitment can also lead to the premature termination of athletic careers during adolescence. Research has consistently indicated that only a small proportion of youth athletes progress to elite-level adult sport (Gulbin et al., 2010). Nevertheless, many young athletes develop a strong athletic identity and may base their future expectations primarily on their sporting role. As a result, the unexpected or forced termination of a sport career may leave athletes insufficiently prepared for life beyond sport, potentially complicating their transition to alternative career pathways and their integration into the labor market (Ryba et al., 2016).

In Hungary, youth sport is typically organized within a well-structured, club or academy-based system that is closely linked to education. Although the concept of dual career has gained increasing attention in recent years, its practical implementation still varies across sports disciplines and sport organizations. As a result, student-athletes often face challenges when trying to balance training and competition with their academic responsibilities (Oláh et al., 2022), especially during secondary school.

Handball, as one of the most popular and successful team sports in the country, operates within a highly organized talent development framework. This makes it a particularly suitable context for examining how sport and education can be balanced during adolescence. The international success of Hungarian handball further highlights this relevance, as both the men's and women's national teams have qualified for the last Olympic Games in Beijing.

### Athlete personality and development

In addition to the developmental demands outlined above, athlete personality represents another important factor in understanding how young athletes approach their future careers. Athlete personality is often characterized by high levels of self-confidence, perceived control, and strong self-efficacy, alongside well-developed self-regulation skills (Feltz et al., 2008). However, these characteristics do not necessarily imply a conscious reflection on life beyond sport. Previous research suggests that athletes are often perceived by their environment as less concerned about their future and less oriented toward long-term career planning (Wylleman et al., 2013).

In this context, curiosity and openness have recently received increasing attention as important psychological dimensions in explaining successful transitions from sport to other life domains. Empirical findings suggest that interests outside sport, exploratory behavior, and openness to learning may function as protective factors during career transitions, supporting adaptive coping and psychological wellbeing (Kuettel et al., 2017; Park et al., 2013).

Building on these considerations, it is particularly important to examine whether athletes' perceived confidence is accompanied by genuine future orientation and conscious career planning, and how development programs within sport organizations contribute to this process. The developmental practices of sport clubs and academies have traditionally been shaped by a performance-oriented approach in which physical and sport-specific development receive primary attention. Particularly in youth sport environments, training systems, feedback mechanisms, and evaluation processes tend to focus mainly on performance-related domains, while psychological, academic, and social development often receive secondary emphasis (Gulbin et al., 2013). Although this performance-focused approach may support short-term sporting success in the sport organizations, it may also increase the risk of developing a one-dimensional athletic identity and limit athletes' adaptive preparation for life beyond sport (Henriksen et al., 2020a).

### Dual career

In line with the developmental and organizational challenges outlined above, the concept of dual career has emerged, emphasizing the coordinated support of athletic careers alongside educational and labor-market pathways. Dual career development represents a key element in the sustainable and responsible development of talented athletes, as it contributes to educational continuity, balanced lifestyle management, and preparation for life after sport (European Commission, 2017). At the same time, combining sport and education may impose substantial demands on young athletes. Managing these parallel commitments often requires advanced psychosocial skills and supportive environments (Stambulova and Wylleman, 2019).

Accordingly, research on dual careers and holistic athlete development consistently highlights the importance of psychological, academic, and social competencies for sustainable sport careers and successful career transitions. Empirical findings suggest that athletes who develop within supportive environments that emphasize multidimensional development tend to demonstrate more balanced lifestyles and more favorable post-sport outcomes (Stambulova and Wylleman, 2019).

At the same time, a gap can still be observed between theoretical recommendations and their practical implementation, particularly in adolescent sport settings, where developmental priorities are not always systematically integrated into everyday practices (De Maio et al., 2025). Over the past decade, increasing attention has been devoted to the long-term development of athletes, especially in relation to the coordination of sport careers with educational and labor-market pathways (Vicari et al., 2025).

Nevertheless, most existing studies tend to focus on adult or elite athletes, or on the transition following sport career termination, while the preparatory phase during adolescence has received comparatively less empirical attention (Martiniello et al., 2025). As a result, there is still limited understanding of how adolescent athletes' sport career aspirations, educational ambitions, and environmental factors interact in shaping career construction and preparation for life both within and beyond sport. In particular, studies focusing on secondary school-aged athletes within specific sport contexts remain scarce.

### Theoretical background

To provide a theoretical foundation for dual career processes, the present study is grounded in career construction theory and the concept of career adaptability, which provides a framework for understanding the interaction between individual and environmental factors within the dual career process (Savickas, 1997; Savickas and Porfeli, 2012). According to this theoretical perspective, career development is not simply the result of a single decision but rather an ongoing process of construction in which individuals shape their future through their goals, self-concepts, perceived competencies, and feedback from their environment (Stambulova and Henriksen, 2025). Success within athletic careers may strengthen athletic identity, sometimes leading to the marginalization of educational or non-sport career planning during a developmental stage when career construction is particularly sensitive (Vidal Vilaplana et al., 2022).

From this perspective, young athletes do not make career-related decisions in isolation but operate within a dynamic system involving sport organizations, coaches, schools, teachers, and family members. Consequently, career construction depends not only on athletes' personal aspirations but also on the learning opportunities and developmental support provided by their environment. The success of a dual career therefore reflects a multi-level process involving cooperation and coordinated support among multiple stakeholders (Henriksen et al., 2020b).

In line with these considerations, adolescence is considered a particularly sensitive stage of career development, during which changes in self-concept and future orientation make career construction a central developmental task (Super, 1980). Career construction theory further emphasizes that individuals actively shape their career trajectories through processes of self-reflection, adaptability, and meaning making (Savickas, 2013). Career aspirations and decisions therefore emerge from the dynamic interaction between personal and environmental influences (Lent et al., 1994).

### Aim of the study

Only a limited number of studies have looked at adolescent athletes' personality characteristics, sport career aspirations, educational ambitions, and environmental support within a single framework. In addition, athletes' and coaches' perspectives are rarely examined together. As a result, there is still a limited understanding of how secondary school athletes prepare for careers both within and beyond sport (Hong et al., 2022).

This study focuses on adolescent handball players, a high-intensity team sport with substantial training demands and a structured development system (Kovács et al., 2024). The relatively high risk of injury and illness (Drole et al., 2025), along with early drop-out (Kassay et al., 2026), further highlights the importance of preparing for life beyond sport, making handball a particularly relevant context for examining dual career processes.

Therefore, the aim of the present study was to explore how adolescent student-athletes' personality traits, sport career aspirations, parallel educational ambitions, and perceived environmental support contribute to their career construction and preparation for life after sport. In addition, coaches were included to examine how they interpret these factors, and which developmental priorities and support practices they consider most relevant for supporting athletes' dual careers.

## Materials and methods

The present study employed a cross-sectional survey design to examine adolescent handball players' and coaches' perceptions of dual career development and the developmental role of sport organizations in Hungary. The research focused on identifying patterns related to athletes' personality characteristics, future orientation, perceived developmental practices within clubs, and the perceived responsibilities of sport organizations in supporting holistic athlete development. By comparing the perspectives of players and coaches, and also genders, the study aimed to provide a broader understanding of how developmental environments in youth sport contribute to dual career preparation.

The study included 507 secondary school-aged handball players (*M* = 16.36 ± 2.19 years; 42.8% male, 57.2% female) and 279 coaches (*M* = 44.60 ± 12.74 years; 57.35% male, 42.65% female). Participants were recruited from all first- and second-division male and female handball clubs in Hungary (*N* = 60), aiming to include the entire target population. All eligible players and their coaches holding a valid competition license were invited to take part. In total, 1,920 players and 480 coaches were contacted, of whom 507 athletes (26.4%) and 279 coaches (58.1%) completed the questionnaire.

The online questionnaire was distributed via official club contacts and coaching networks, ensuring broad national coverage. Data collection was based on the Erasmus+ DC4AC questionnaire, which included background variables and 23 items rated on a four-point Likert scale (1 = strongly disagree, 4 = strongly agree). The questionnaire assessed athletes' personality characteristics, perceptions of developmental practices within clubs and sport departments, and respondents' views regarding the responsibility of sport organizations for different areas of athlete development. Internal consistency was assessed using Cronbach's alpha, which indicated good reliability for the overall sample (α = 0.85) as well as for both subgroups (players: α = 0.85; coaches: α = 0.86).

Although the Shapiro–Wilk test was significant (*p* < 0.05), this is likely due to the large sample size, which can make normality tests overly sensitive (Ghasemi and Zahediasl, 2012). Skewness and kurtosis values were within acceptable ranges, and Q–Q plots suggested approximate normality; therefore, parametric tests were applied.

Descriptive statistics were used to describe sample characteristics and response distributions. Differences between athletes and coaches were examined using independent samples *t*-tests. All analyses were conducted in SPSS 26.0, with the level of significance set at *p* < 0.05.

The study was conducted in accordance with the principles of the Declaration of World Medical Association (2003). Written informed consent was obtained from the parents or legal guardians of all participating minors prior to data collection. Ethical approval for the study was granted by the Institutional Review Board of Eszterházy Károly Catholic University (Approval ID: RK/1585/2025).

## Results

According to self-reports, 94.0% of the total sample indicated that they were familiar with the concept of dual career. In addition, 64.9% of the athletes reported that they had already encountered situations in which they had to choose between their athletic career and their studies.

Based on the responses of the total sample, both athletes and coaches generally perceived the athletes as curious (*M* = 3.070 ± 0.895). In contrast, the overall assessment suggests that athletes show lower levels of concern regarding their dual careers (*M* = 2.089 ± 0.896) and also their future (*M* = 1.888 ± 0.902) (Table 1).

**Table 1:** Athletes' personality characteristics and future plans (Likert scale 1–4).

Perceived characteristics of athletes' personality and future plans	Mean	SD	Min–Max	CI
In your opinion, to what extent do players have a curious nature?	3.070	0.895	1–4	3,007–3,132
To what extent do players think about what they will do after their athletic careers?	2.891	0.895	1–4	2,827–2,953
To what extent do players feel prepared for life after sport?	2.832	0.837	1–4	2,773–2,890
In your opinion, to what extent do players feel that they control the direction of their lives?	2.794	0.856	1–4	2,733–2,853
In your opinion, how confident are the players?	2.733	0.879	1–4	2,671–2,794
In your opinion, how much do players worry about their dual career?	2.089	0.896	1–4	2,026–2,151
In your opinion, how much do players worry about their future?	1.888	0.902	1–4	1,824–1,951

CI, Confidence interval (−95%−95%); Cronbach α, 568.

Based on the overall responses, the existing developmental and support practices of sport organizations are primarily oriented toward physical development (*M* = 3.474 ± 0.682) and sport-specific development (*M* = 3.276 ± 0.773). In contrast, academic/intellectual development (*M* = 2.760 ± 0.872) and psychological/emotional development (*M* = 2.678 ± 0.948) receive the least emphasis (Table 2).

**Table 2:** To what extent does the department, club, or academy that you belong to address the development of the following areas? (Likert scale 1–4).

What extent is the sport organization address the development in these areas?	Mean	SD	Min–Max	CI
Physical development	3,474	0,682	1–4	3,4267–3,522
Sport-specific development	3,276	0,773	1–4	3,221–3,330
Personal development	3,209	0,801	1–4	3,153–3,266
Moral development	2,956	0,897	1–4	2,893–3,019
Social development	2,930	0,855	1–4	2,870–2,989
Communication development	2,912	0,907	1–4	2,848–2,975
Academic/intellectual development	2,760	0,872	1–4	2,699–2,821
Psychological/emotional development	2,678	0,948	1–4	2,611–2,744

CI, Confidence interval (−95%−95%); Cronbach α, 901.

In relation to the perceived developmental responsibilities of sport clubs and departments, respondents attributed the greatest responsibility to these institutions for sport-specific development (*M* = 3.643 ± 0.577) and physical development (*M* = 3.431 ± 0.766). The lowest levels of perceived responsibility were reported for communication development (*M* = 2.998 ± 0.831) and academic/intellectual development (*M* = 2.720 ± 0.891) (Table 3).

**Table 3:** In your opinion, to what extent does the sports organization or academy that you belong to is responsible for the development of the following areas? (Likert scale 1–4).

What extent is the sport organization is responsible for the following developmental areas?	Mean	SD	Min–Max	CI
Sport-specific development	3,643	0,577	1–4	3,603–3,684
Physical development	3,431	0,766	1–4	3,377–3,484
Personal development	3,278	0,770	1–4	3,224–3,332
Moral development	3,059	0,828	1–4	3,001–3,117
Psychological/emotional development	3,029	0,804	1–4	2,972–3,085
Social development	3,017	0,841	1–4	2,958–3,076
Communication development	2,998	0,831	1–4	2,940–3,056
Academic/intellectual development	2,720	0,891	1–4	2,657–2,782

CI, Confidence interval (−95%−95%); Cronbach α, 876.

### Perspectives of athletes and coaches

Athletes reported that they feel more prepared for life after sport (*t* = −7.083), think more frequently about what they will do after their athletic careers (*t* = 6.538), perceive themselves as having greater control over their lives (*t* = −4.181), and consider themselves more curious (*t* = 3.738) than coaches perceived them to be (all *p* < 0.05). They also reported experiencing stronger existing personal development practices within their clubs and sport departments (*t* = 2.103) than coaches reported.

In contrast, coaches placed greater emphasis on several developmental areas than athletes did. In their view, psychological/emotional (*t* = −3.468), academic/intellectual (*t* = −2.636), and moral development (*t* = −2.421) received more attention within clubs than athletes perceived (all *p* < 0.05). Coaches also indicated that a wide range of areas—including personal, psychological/emotional, social, academic/intellectual, sport-specific, moral, and communication development—should receive greater emphasis than is currently perceived by athletes (all *p* < 0.05).

### Gender differences

Female athletes reported that they think more frequently about what they will do after their athletic careers (*t* = −2.904), show greater concern about their future (*t* = −2.392) and their dual career (*t* = −2.337), and consider themselves more curious (*t* = −3.699) than their male counterparts (all *p* < 0.05). In contrast, male athletes reported higher levels of self-confidence than female athletes (*t* = 4.175; *p* < 0.05). Female athletes also perceived that their clubs and sport departments place greater emphasis on physical development (*t* = −2.972), social development (*t* = −3.573), sport-specific development (*t* = −3.082), and moral development (*t* = −2.775) compared with male athletes (all *p* < 0.05). Similarly, female athletes attributed greater responsibility to clubs and sport departments for physical development (*t* = −3.909), psychological/emotional development (*t* = −2.730), social development (*t* = −2.722), academic/intellectual development (*t* = −2.416), sport-specific development (*t* = −3.737), moral development (*t* = −3.112), and communication development (*t* = −2.074) compared with male athletes (all *p* < 0.05).

Considering coaches' gender, male coaches were more likely than female coaches to believe that athletes manage their lives adequately (*t* = 3.703, *p* < 0.05). In contrast, female coaches perceived a stronger emphasis within clubs and sport departments on psychological/emotional development (*t* = −2.672), social development (*t* = −2.223), and communication development (*t* = −2.784) compared with their male counterparts. Furthermore, female coaches attributed greater responsibility to clubs and sport departments in supporting athletes' psychological/emotional development (*t* = −1.962), academic/intellectual development (*t* = −2.384), and communication development (*t* = −2.698) than male coaches (all *p* < 0.05).

## Discussion

The present study aimed to explore how adolescent handball players and their coaches perceive dual career preparation and the developmental role of sport organizations. Overall, the results suggest that although awareness of the dual career concept is relatively high, many athletes already experience conflicts between sport and education, while showing relatively low levels of concern about their future and dual career pathways. This may indicate that dual career is recognized at a general level but is not yet fully integrated into athletes' long-term thinking. From the perspective of career construction theory, this pattern may reflect a developmental stage in which future-oriented thinking and career adaptability are still evolving rather than fully established (Savickas, 2013; Super, 1990).

At the same time, the results point to a clear imbalance in the developmental orientation of sport organizations. Both athletes and coaches perceived that physical and sport-specific development dominate current practices, whereas psychological, academic, and communication-related domains receive less attention. This finding is consistent with previous research highlighting the gap between theoretical models of holistic athlete development and their practical implementation (Brown et al., 2018; Henriksen et al., 2020a). From the perspective of career adaptability, which includes dimensions such as concern, control, curiosity, and confidence (Savickas, 2013), this imbalance is particularly relevant. While the relatively higher levels of perceived curiosity and confidence may support adaptability, the low levels of future concern suggest that one of its key components remains underdeveloped.

A similar tension is reflected in the discrepancy between perceived practices and expectations. Although respondents attributed considerable responsibility to sport organizations for supporting broader developmental areas, these domains appear to be less systematically addressed in everyday practice. This may indicate that performance-oriented environments continue to prioritize short-term sporting success over long-term career construction processes. Such a tendency aligns with earlier findings emphasizing the dominance of athletic identity within talent development systems (Stambulova et al., 2009, 2024).

Differences between athletes' and coaches' perspectives provide further insight into this issue. Athletes tended to evaluate their own preparedness and developmental context more positively, whereas coaches emphasized the need for stronger support across multiple domains. This discrepancy may reflect differences in experience and role expectations. At the same time, it may also suggest that athletes' self-perceptions are shaped by a strong athletic identity, which can limit critical reflection on future career challenges beyond sport (Ryba et al., 2015a).

Gender differences also emerged as an important aspect of the findings. Female athletes and female coaches appeared to demonstrate greater sensitivity toward future planning and psychosocial aspects of development, whereas male athletes reported higher levels of self-confidence. These patterns may indicate differences in how career adaptability resources are activated across genders. In particular, greater concern and reflection among female participants may support more proactive career construction, which is consistent with previous findings on gender-related differences in developmental perspectives (Wylleman et al., 2004).

Taken together, the findings highlight a central dilemma in adolescent sport development, which is the strong emphasis on athletic identity within performance-oriented environments. While such environments may effectively support short-term sporting success, they may also limit the development of broader identity structures and future orientation. According to career construction theory, identity formation and adaptability are closely interconnected processes, and the dominance of a single role may constrain the development of alternative career pathways (Savickas, 2013). This may help explain why athletes report relatively low concern about their future despite being embedded in highly structured developmental systems (López-Flores et al., 2020).

The findings of the present study are also in line with international research suggesting that athletes often deprioritize career planning outside sport during their active careers (Hernando Domingo et al., 2025; Quinaud et al., 2022). This tendency is particularly pronounced when the athletic role constitutes a central component of self-concept (Ryba et al., 2015b).

From a practical perspective, the results suggest that the developmental approach in secondary school talent development sport may benefit from a more balanced and intentional focus. In addition to physical and sport-specific development, greater emphasis could be placed on psychological, academic, and life-management competencies. Strengthening these areas may support the development of career adaptability and encourage more conscious engagement with future career planning. In this process, coaches should be considered not only as technical instructors but also as facilitators of holistic development, which requires appropriate training and institutional support.

Future research could further explore these processes using longitudinal designs to better understand how career adaptability, identity development, and environmental factors interact over time. In addition, qualitative approaches may provide deeper insight into how athletes and coaches interpret dual career challenges in everyday practice. Finally, comparative studies across different education, sports and cultural contexts would help to clarify the generalizability of the present findings.

## Limitations

Although the study provides useful insights, some limitations should be considered. First, all data were self-reported, which may introduce common method bias, for example through subjective interpretations or social desirability. In addition, the cross-sectional design does not allow for causal conclusions about the relationships between athletes' perceptions, developmental environments, and dual career preparation. The study also focused specifically on adolescent handball players within a particular talent development context, so the findings may not be directly transferable to other sports, age groups, or settings. Moreover, as the research was conducted in Hungary, the characteristics of the national sport and education system may have influenced the results.

## Conclusion

This study demonstrates that although young handball players are generally aware of the dual career concept, it is not yet a central part of how they think about their future and long-term planning. Sport environments in youth handball still focus mainly on performance, while areas like education and personal development receive less attention. This may make it harder for handball players to prepare for life beyond competitive sport. The results highlight the need for a more balanced approach, where coaches and sport organizations actively support not only athletic success but also athletes' long-term career planning.

## Data Availability

The original contributions presented in the study are included in the article/supplementary material, further inquiries can be directed to the corresponding author.
